# A small protein probe for correlated microscopy of endogenous proteins

**DOI:** 10.1007/s00418-018-1632-6

**Published:** 2018-01-11

**Authors:** Marit A. de Beer, Jeroen Kuipers, Paul M. P. van Bergen en Henegouwen, Ben N. G. Giepmans

**Affiliations:** 1 Department of Cell Biology, University of Groningen, University Medical Center Groningen, Groningen, The Netherlands; 2 Division of Cell Biology, Department of Biology, Faculty of Science, Utrecht University, Utrecht, The Netherlands

**Keywords:** Probes, Correlated microscopy, FLIPPER, Nanobody, APEX2

## Abstract

Probes are essential to visualize proteins in their cellular environment, both using light microscopy as well as electron microscopy (EM). Correlated light microscopy and electron microscopy (CLEM) requires probes that can be imaged simultaneously by both optical and electron-dense signals. Existing combinatorial probes often have impaired efficiency, need ectopic expression as a fusion protein, or do not target endogenous proteins. Here, we present FLIPPER-bodies to label endogenous proteins for CLEM. Fluorescent Indicator and Peroxidase for Precipitation with EM Resolution (FLIPPER), the combination of a fluorescent protein and a peroxidase, is fused to a nanobody against a target of interest. The modular nature of these probes allows an easy exchange of components to change its target or color. A general FLIPPER-body targeting GFP highlights histone2B-GFP both in fluorescence and in EM. Similarly, endogenous EGF receptors and HER2 are visualized at nm-scale resolution in ultrastructural context. The small and flexible FLIPPER-body outperforms IgG-based immuno-labeling, likely by better reaching the epitopes. Given the modular domains and possibilities of nanobody generation for other targets, FLIPPER-bodies have high potential to become a universal tool to identify proteins in immuno-CLEM with increased sensitivity compared to current approaches.

**Electronic supplementary material:**

The online version of this article (10.1007/s00418-018-1632-6) contains supplementary material, which is available to authorized users.

## Introduction

An ultimate goal in microscopy is to detect targets at both the fluorescent level, opening the possibilities to study live cells and investigate large areas, as well as detection using electron microscopy (EM) to allow localization with molecular precision (nanometer range) in the context of ultra-structure. These benefits are all combined in correlated light microscopy (LM) and EM [CLEM; reviewed in (de Boer et al. 2015; Loussert Fonta and Humbel 2015)]. In CLEM probes, both the fluorescent and electron-dense signal need to be specifically targeted to the protein of interest.

**Fig. 1:**
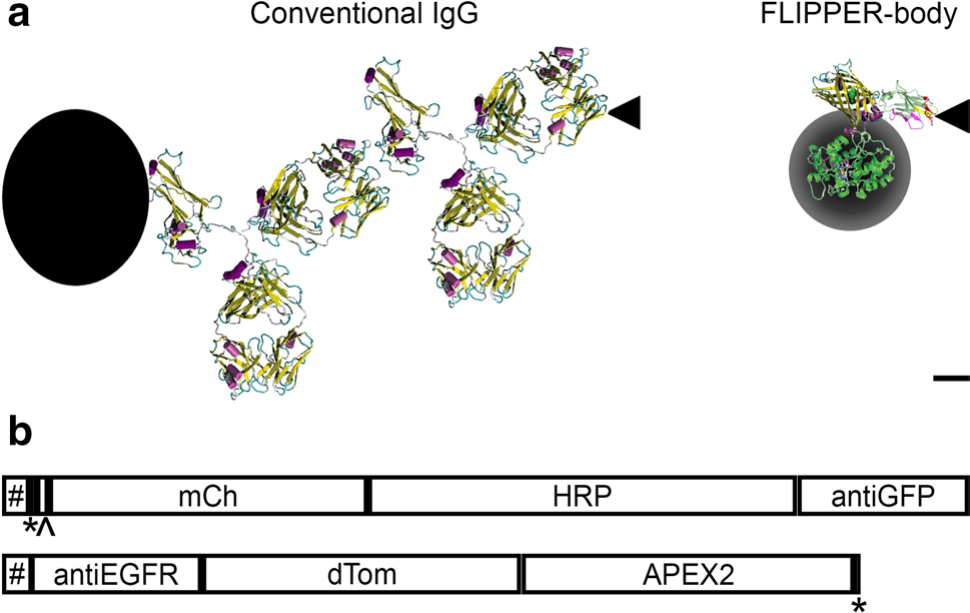
Small FLIPPER-bodies for single-step CLEM labeling. **a** Left: traditional indirect antibody labeling conjugated with QD, represented by black dot. Right: a single-labeling step with FLIPPER-body. Black circle represents DAB conversion. Black triangle: antigen. Bar 2 nm. Adapted from (Beghein and Gettemans 2017; Giepmans et al. 2006; Martell et al. 2012). **b** Boxes representing the modules of the anti-GFP and anti-EGFR FLIPPER-bodies. The boxes are shown in proportion to each other. Signal peptide (#), His6-tag (*), thrombin cleavage site (^), mCherry (mCh), dTomato (dTom), horseradish peroxidase (HRP), ascorbate peroxidase 2 (APEX2), anti-GFP nanobody (anti-GFP), anti-EGFR nanobody (anti-EGFR)

Specific probe targeting for endogenous proteins is usually done with antibodies, which can be applied before (pre-embedding) or after EM preparation (post-embedding). The advantage of post-embedding labeling is the preserved ultra-structure, but antigen binding is impaired due to EM preparation. Pre-embedding labeling has a compromised ultra-structure partly as a result of the permeabilization, but a good accessibility to the antigens (de Boer et al. 2015; Schnell et al. 2012). Another disadvantage of antibodies is their size: 150 kDa with a length of 14 nm for typical IgG (Fig. 1a). This size results in a distance up to 28 nm between target and label (Mikhaylova et al. 2015; Muyldermans 2013).

Nanobody-mediated targeting reduces the distance of target and probe. Nanobodies are the smallest antigen-binding proteins that are derived from heavy-chain antibodies of *Camelidea* species, consisting of a single chain, as opposed to IgGs (Hamers-Casterman et al. 1993; Helma et al. 2015). Genetic fusion of nanobodies with fluorescent proteins [FPs; chromobodies (Rothbauer et al. 2006)], as well as with ascorbate peroxidase 2 (APEX2) for EM visualization (Ariotti et al. 2015) have been used in co-expression systems and successfully show identification of several targets. However, co-expression in cells also affects protein function for some targets [(Helma et al. 2015); see below]. Indirect EM labeling using an anti-nanobody antibody and a protein-A conjugated to gold particles was recently pioneered, but still results in a relatively large distance, > 17 nm, from the gold particle to the target protein (HER2) (Kijanka et al. 2017). We reasoned that a single multi-domain protein that (1) can specifically target proteins based on a nanobody; (2) directly visualizes these using fluorescent proteins; and (3) identifies targets in EM using peroxidase-mediated DAB precipitation may have better labeling efficiency than particle-based detection in EM. Since the architecture of the genetically-encoded probes allows easy domain swapping, virtually unlimited combinations between targeting modules, FPs and peroxidase can be made (Fig. 1b). Note that, as opposed to most current genetically-encoded probes, our FLIPPER-bodies are first secreted by producer cells and subsequently used, either as a supernatant or purified, like hybridoma generated monoclonal antibodies. Our results show that this protein-based probe highly efficiently labels targets, including in difficult to reach subcellular areas, and outperforms current available alternatives.

## Methods

### Plasmids

The sequences of the used FLIPPER-bodies are presented (Supplementary data). The backbones of the FLIPPER-bodies were ordered by Eurofins Genomics (Ebersberg, Germany) and modules were switched with standard molecular cloning tools. The signal peptide originated from EpCAM. The cDNA of mCherry and dTomato [gifts from Roger Tsien; (Shaner et al. 2004)] were inserted using *Bam*HI and *Eco*RI. Horseradish peroxidase (HRP) was replaced by APEX2 [gift from Alice Ting: Addgene plasmid # 49385; (Lam et al. 2015)] using *Eco*RI and *Not*I. Anti-EGFR (epidermal growth factor receptor) nanobody was replaced by anti-HER2 (Human Epidermal growth factor Receptor 2) using *Age*I and *Eco*RI. Other plasmids used were EGFR-GFP [gift from Alexander Sorkin: Addgene plasmid # 32751; (Carter and Sorkin 1998)], Histone2B (H2B)-GFP [gift from Geoff Wahl: Addgene plasmid # 11680 (Kanda et al. 1998)] and SP-His_6_-GPI-CFP (Hauser and Tsien 2007).

### Cell culture and transfection

HEK293T, A431, and SkBr3 cells were cultured in DMEM (Invitrogen), and CHO-K1 cells were cultured in DMEM/Ham’s F-12 1:1 (Lonza), all supplemented with 5% Fetal Bovine Serum (FBS, Greiner) and penicillin/streptomycin (PAA) at 37 °C in the presence of 5% CO_2_. Cells were transfected using 1:4 DNA/polyethylenimine 1 mg/ml (PEI MW 25,000, Polysciences). Stable CHO-K1 cell lines producing anti-GFP and anti-EGFR FLIPPER-bodies were selected using 1 mg/ml G418 (Invitrogen) and subsequent FACS-sorted (MoFlo Astrios, Central Flowcytometry Unit, UMCG). For imaging, HEK293T and A431 cells were grown on glass bottom dishes (Greiner); SkBr3 cells were grown on gridded glass bottom dishes (MatTek).

### Fixation

The cells were fixed by adding dropwise an equal volume of 2% paraformaldehyde/0.2% glutaraldehyde in 0.1 M sodium cacodylate buffer pH 7.4 to the medium. After 10 min, the medium/fixative was replaced by the fixative described above for 30 min and rinsed once 5 min with 0.1 M sodium cacodylate buffer and twice 5 min with Phosphate Buffered Saline (PBS) pH 7.3. HEK293T expressing H2B-GFP was permeabilized with 0.1% Triton X-100 in PBS for 7 min and washed three times 5 min with PBS. All steps were performed at R.T.

### FLIPPER-body production

FLIPPER-bodies are produced and secreted by stable CHO cells or by transiently transfected HEK293T cells. Conditioned media were collected after 2 days of culturing. The activity of the secreted peroxidase was tested as a 1:1 mixture of medium and diaminobenzidine (DAB, 5 mg DAB in 10 ml PBS, filtered through Whatman 0.2 μm filter with an additional 3 μl of 30% H_2_O_2_).

### Antibodies

Primary antibodies used were rabbit anti-GFP (Rockland, 600-401-215, 1:200) and mouse anti-EGFR (Santa Cruz, sc120, 1:200). Secondary antibodies used were goat anti-mouse Alexa Fluor 546 (Invitrogen, A11030, 1:500), donkey anti-rabbit Alexa Fluor 594 (Invitrogen, A21207, 1:500), goat anti-mouse quantum dot (QD) 655 (Life Technologies, Q11021MP, 1:500), goat anti-rabbit QD655 (Life technologies, Q11421MP, 1:500), sheep anti-mouse HRP (GE-Healthcare, NA931V, 1:500), donkey anti-rabbit HRP (GE-Healthcare, NA934V, 1:500), goat anti-mouse 10 nm gold (BBI solutions, EM.GMHL10, 1:50), and goat anti-rabbit 10 nm gold (BBI solutions, EM.GAR10, 1:50).

### Immuno-labeling, confocal microscopy

HEK293T, A431, and SkBr3 cells were labeled with FLIPPER-bodies for 45 min and rinsed twice 5 min with PBS. Primary and secondary antibodies were diluted in 1% BSA/PBS and followed by rinsing three times with PBS. Next, the cells were incubated with Hoechst (Sigma-Aldrich, 861405, 1 µg/ml), for 10 min, except H2B-GFP labeled with QDs, and rinsed three times 5 min with PBS. All steps were performed at room temperature. Fluorescent images were generated using confocal imaging (Zeiss LSM 780, Plan-Neofluar 63×/N.A. 1.3 Imm Korr DIC M27 lens).

### DAB polymerization, embedding, electron microscopy

DAB (5 mg DAB in 10 ml PBS, filtered through Whatman 0.2 μm filter with an additional 3 μl of 30% H_2_O_2_) was added to the cells for 15 min. Afterward, the cells were rinsed twice for 5 min with PBS. Samples were prepared for EM as described previously (Kuipers et al. 2015b). In short, the cells were post-fixed with 1% OsO_4_ in 0.1 M sodium cacodylate buffer and dehydrated with an increasing ethanol series. Finally, the cells were embedded in EPON. The glass bottom was removed using hydrogen fluoride for 10 min.

Ultrathin sections (80 nm) were obtained and collected at 150 mesh copper grids. Sections were not contrasted with uranyl or lead. The samples were pre-irradiated using FEI CM100 TEM to stabilize them in the electron beam and images were recorded using a Zeiss Supra55 scanning microscope (Kuipers et al. 2015a; Sokol et al. 2015). STEM images were taken with 2.5 nm pixel size at 28 KV. All data are available at http://www.nanotomy.org.

### CLEM workflow

For all experiments, cells were fixed and labeled, either with or without permeabilization. Fluorescence microscopy was performed at the wet, fixed samples. Subsequently, the DAB precipitates were formed and cells were processed for EM, using osmium fixation and epon embedding. While this procedure leads to loss of fluorescence, reviewed in de Boer et al. (2015), the FLIPPER-bodies allow identification of the targets at the nanometer-range scale by analysis of osmiophilic DAB polymers. For a more complete flow chart of alternative labeling procures, see de Boer et al. (2015).

## Results

Previously, we introduced the combination of FP and HRP linked to a protein of interest as a probe for CLEM inside cells, named FLIPPER (Kuipers et al. 2015b). In addition, APEX2 (Lam et al. 2015) is used to generate the EM-detectable signal. FLIPPER-bodies are multi-modular fusions between a nanobody, an FP and a peroxidase (Fig. 1b). Because of the inclusion of a signal peptide, the FLIPPER-body is secreted by producer cells and the conditioned media is used to label other cells. Alternatively, using the included His-tag, the FLIPPER-bodies can be purified. The different FLIPPER-bodies below were all produced by mammalian cells. As a first test for expression and activity, the medium was simply tested for DAB conversion (Fig. S1).

### FLIPPER-bodies visualize nucleus-localized GFP at the LM and EM levels

**Fig. 2:**
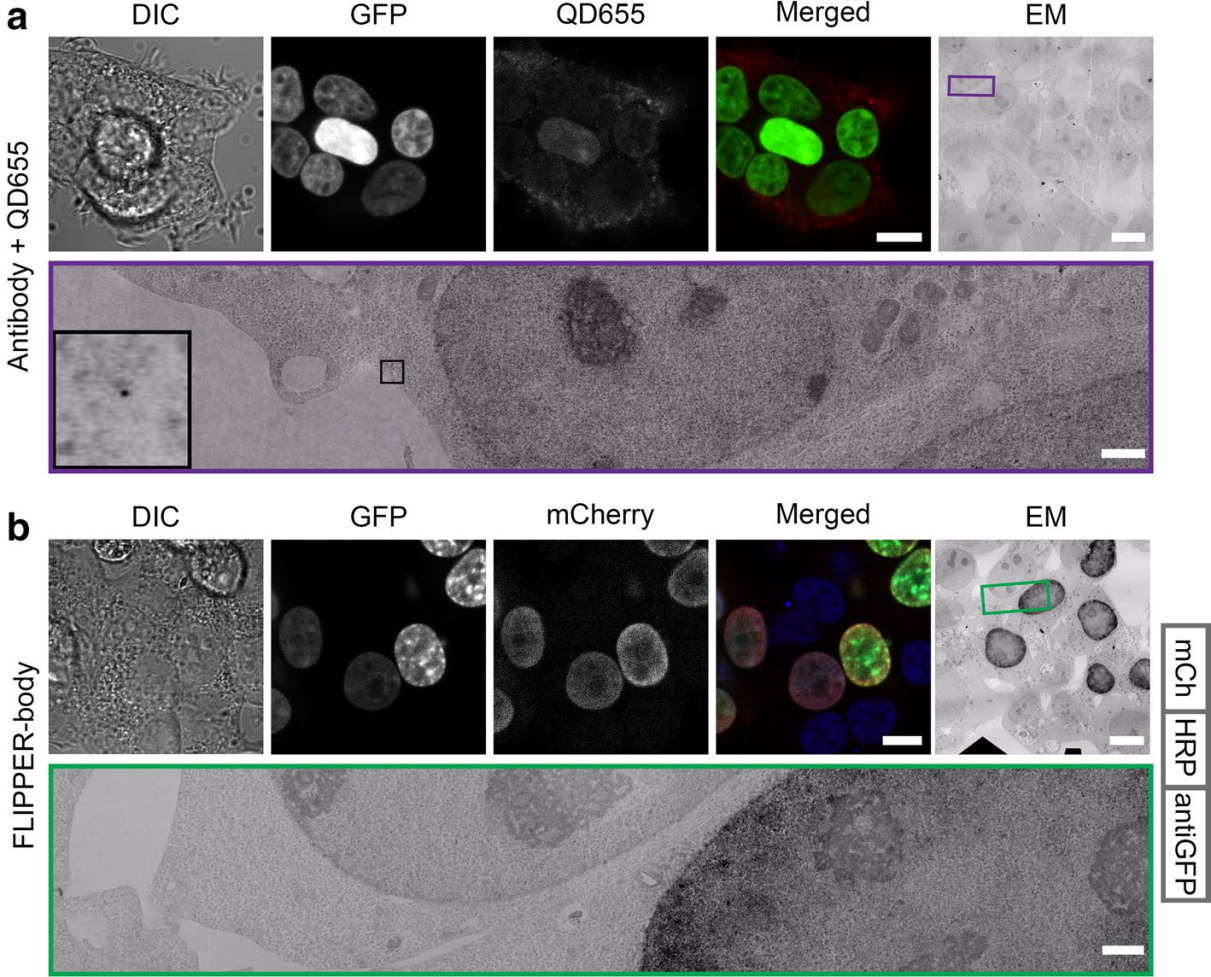
FLIPPER-body labeling of nuclear localized GFP. HEK293T cells expressing H2B-GFP. **a** H2B-GFP was detected by indirect labeling with rabbit anti-GFP and secondary IgG conjugated to QD655. Note that fluorescence detection of QDs is limited to the cytoplasm, which may be non-specific labeling. High-resolution EM images also show only QDs in the cytoplasm. **b** H2B-GFP was detected by direct labeling with FLIPPER-bodies containing mCherry. The used FLIPPER-body is shown at the right. Note the specificity of the mCherry signal in only the GFP positive cells. With EM, the black DAB precipitates are seen in the nuclei. *DIC* differential interference contrast, *GFP* GFP fluorescence, *QD655* QD655 fluorescence, *mCherry* FLIPPER-body, *merged* GFP and mCherry, *EM* ultrathin EM section. Bars LM and EM 10 µm, EM zoom in 1 µm. Unbiased large-scale high-resolution EM images are available via http://www.nanotomy.org

As a proof-of-concept, a FLIPPER-body was created consisting of a nanobody targeting GFP fused to mCherry and HRP. After fixation and permeabilization H2B-GFP positive cells were labeled directly with an anti-GFP FLIPPER-body only or as a control an anti-GFP IgG that was detected with a secondary antibody conjugated to QD655, another CLEM probe for immuno-labeling [(Nisman et al. 2004; Giepmans et al. 2005); Fig. 2]. H2B-GFP expression is clearly visible in the nucleus, while the limited penetration of the QDs can be seen by detection of some QDs in the cytoplasm and not in the nucleus (Fig. 2a). None of the other secondary antibodies conjugated to either Alexa594, HRP, or 10-nm gold particles could be discerned in the nucleus either (Fig. S2). In contrast, the FLIPPER-body labeling shows in GFP positive nuclei both a mCherry signal and the electron-dense signal derived from DAB conversion (Fig. 2b). Note that the signal is found in the H2B-GFP-transfected cells only. These results demonstrate the functionality of FLIPPER-bodies for intranuclear labeling of proteins, as well as the feasibility to target GFP. Therefore, we set out if we can use FLIPPER-bodies as generic tools, starting with targeting a cell surface receptor.

### Specific labeling of the EGFR by FLIPPER-bodies

EGFR was chosen as the second target to optimize and verify FLIPPER-body technology. The new probe, consisting of a well-characterized anti-EGFR nanobody (7D12; Roovers et al. 2011), was positioned at the N-terminal of the FP and APEX2 modules (Fig. 1b). We switched the order, because binding capacity of the nanobody was lost after C-terminal conjugation. dTomato was chosen as FP because of its dimer-forming capacity that may double the fluorescence and EM signals.

**Fig. 3:**
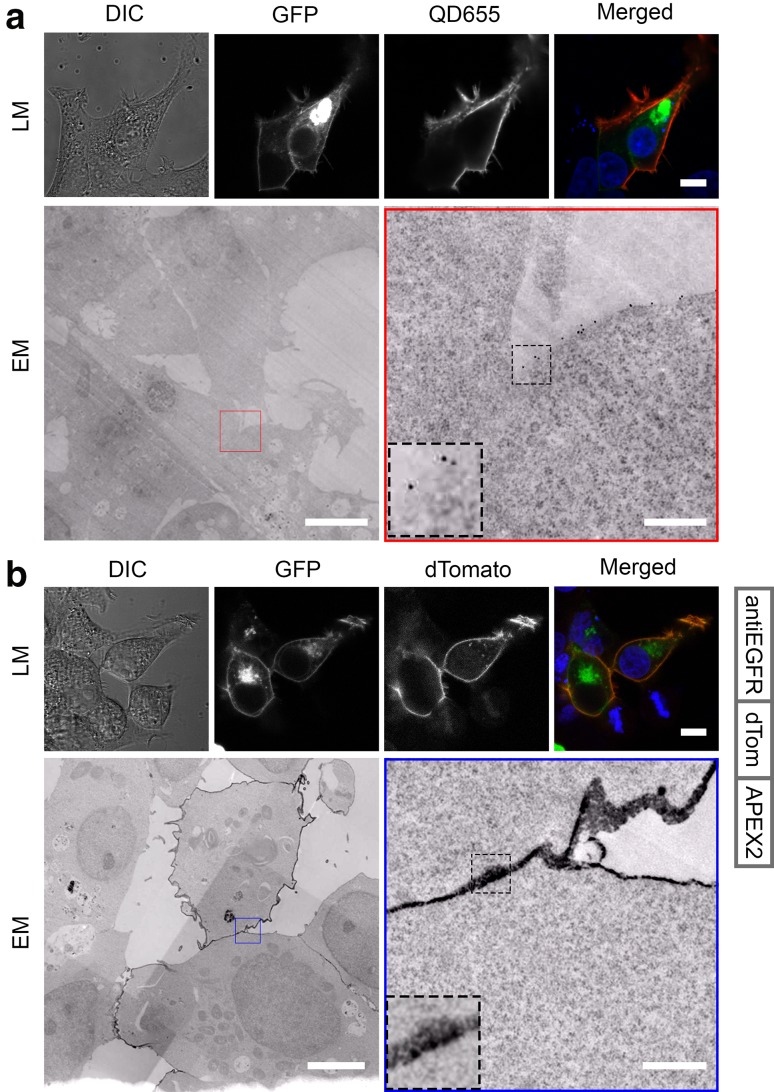
FLIPPER-body targets specific overexpressed EGFR. HEK293T expressing EGFR-GFP. **a** Detection of EGFR by GFP fluorescence, as well as by indirect labeling of EGFR with secondary antibodies conjugated to QD655. Note that QD655 are detected at the periphery of the cells, but not at cell–cell contact sites. **b** Detection of EGFR using FLIPPER-body (cartoon right). The FLIPPER-body labels uniquely the EGFR-GFP positive cells, including at the cell–cell contact sites. *DIC* differential interference contrast, *GFP* GFP fluorescence, *QD655* QD655 fluorescence, *dTomato* FLIPPER-body, *merged* GFP and label, *EM* ultrathin EM section. Bars LM 10 µm, EM 5 µm, EM zoom in 0.5 µm. High-resolution EM images are available via http://www.nanotomy.org

EGFR-GFP overexpressing cells were targeted with traditional immuno-CLEM using IgGs and QDs as described above, as well as with anti-EGFR FLIPPER-body by pre-embedding labeling using non-permeabilized cells. The EGFR-GFP signal is present in the Golgi system as well as on the plasma membrane of the cells (Fig. 3). Traditional labeling clearly revealed QD fluorescence at the apical plasma membrane, but significantly less at cell–cell contact sites (Fig. 3a). Secondary antibodies conjugated with other labels showed similar results (Fig. S3). FLIPPER-body-mediated labeling of EGFR was clearly visible by the dTomato signal as well as by the electron-dense staining (Fig. 3b). Note that the signal was distributed evenly over the surface of the cells, including at sites of cell–cell contacts that were not visualized with the traditional methods.

### Endogenous detection of EGFR

**Fig. 4:**
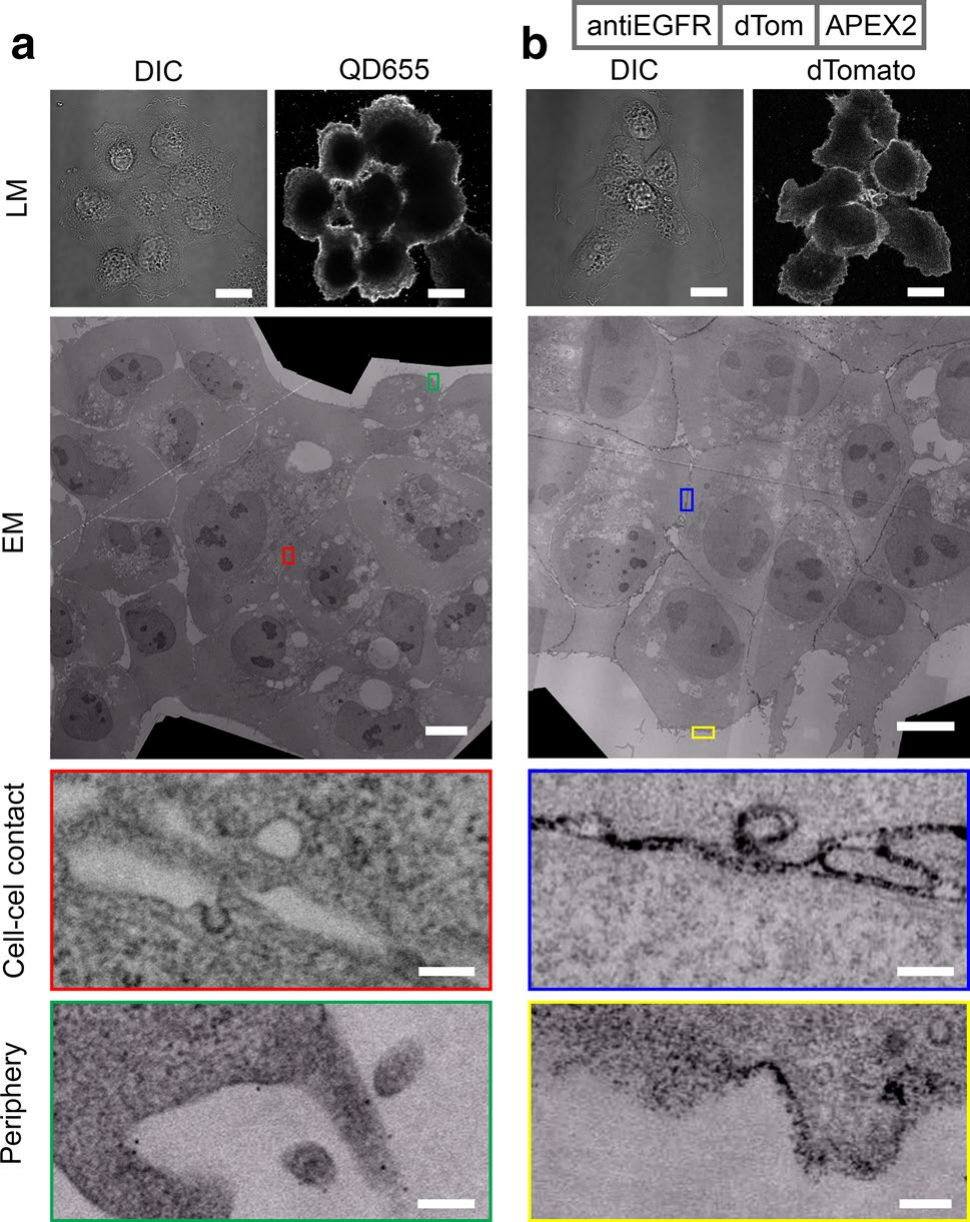
FLIPPER-body reveals localization of endogenous EGFR. **a** EGFR on A431 cell was labeled using anti-EGFR and secondary IgG conjugated with QD655. Note that the QD655 labeling is mainly restricted to the periphery of the cell clusters. **b** EGFR is labeled with FLIPPER-body shown at the top. The cell periphery as well as the cell–cell contact sites are labeled with fluorescence and black staining in EM. *DIC* differential interference contrast, *QD655* QD655 fluorescence, *dTomato* FLIPPER-body. Bars LM 20 µm, EM 10 µm and EM zoom in 200 nm. High-resolution large-scale EM images are available via http://nanotomy.org

Next, we used A431 cells, known for its high EGFR levels (Haigler et al. 1978), to investigate our FLIPPER-body technology for detection of endogenous proteins. The A431 cells were fixed and labeled with the different probes. Indirect labeling of EGFR using QDs resulted in a labeling of the periphery of the cell clusters, while the cell–cell contacts were devoid of any labeling (Fig. 4a). The decreased labeling efficiency at cell–cell contact sites was also seen using IgGs with other labels, even with the small moiety Alexa546 (Fig. S4). Labeling efficiency of the EGFR was much higher with anti-EGFR FLIPPER-body (Fig. 4b). Not only the periphery of the cell clusters was labeled with high efficiency using as assessed by dTomato fluorescence and electron-dense precipitates for LM and EM, respectively, but also the cell–cell contacts sites also have efficient labeling.

### A third target: HER2 detection with correlated microscopy

**Fig. 5:**
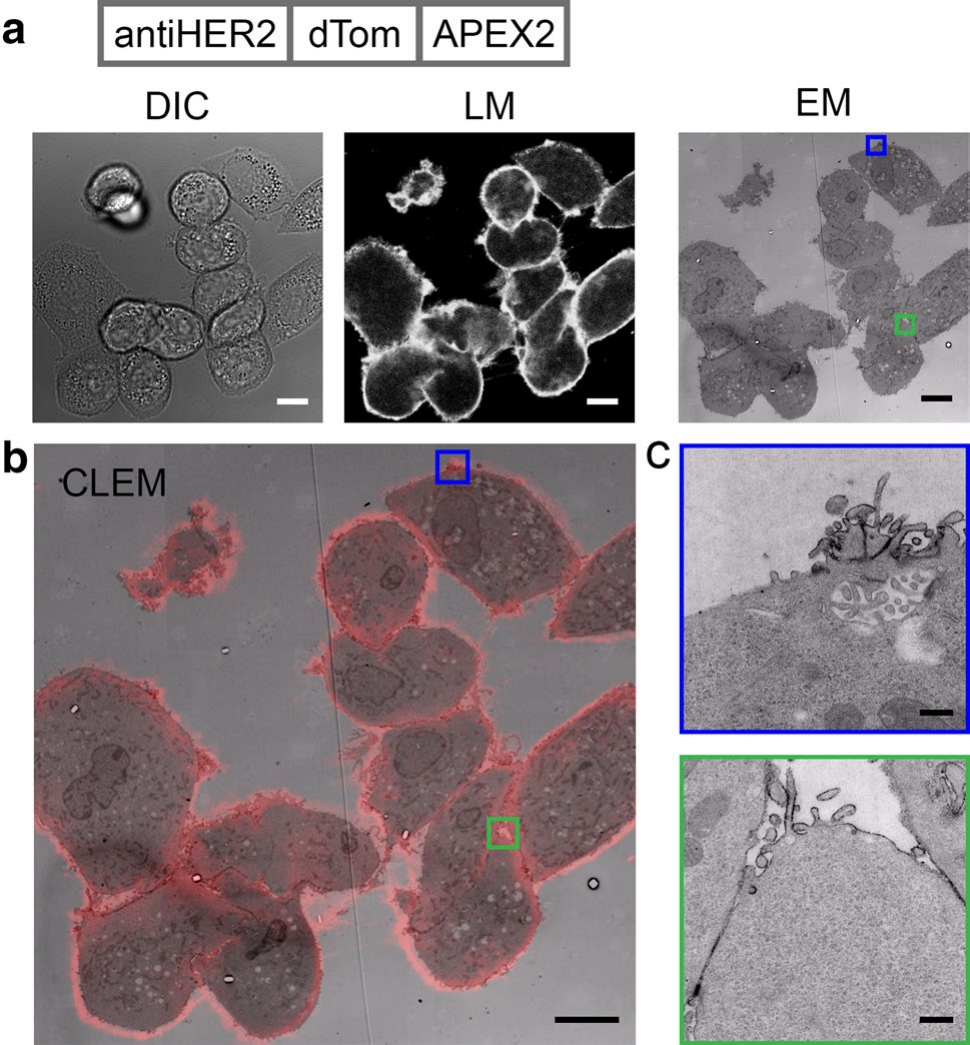
Correlated microscopy of endogenous HER2. SkBr3 cells labeled and processed for CLEM. **a** HER2 detection with anti-HER2 FLIPPER-body with dTomato fluorescence. In EM, a black substrate is surrounding the cells. **b** Overlap of the fluorescence and EM image resulting in the CLEM image. **c** Zoom in EM images shows the labeling at the cells periphery and cell–cell contact sites (green zoom in). It is also noted that there is a heterogeneous distribution of the HER2 labeling (blue zoom in). *DIC* differential interference contrast, *LM* dTomato fluorescence, *EM* ultrathin EM section. Bars LM 10 µm, EM 10 µm and EM zoom in 500 nm. High-resolution EM images are available via http://www.nanotomy.org

FLIPPER-bodies targeting HER2 were created by switching the anti-EGFR nanobody for an anti-HER2 nanobody (11A4). The HER2 nanobody was well characterized in binding capacity and specificity (Kijanka et al. 2013). HER2 is known for its high expression level in breast cancer (Yarden 2001); therefore, the breast cancer cell line SkBr3 was labeled. HER2 detection with FLIPPER-bodies shows high label efficiency in both modalities (Fig. 5a), as emphasized in the CLEM overlay (Fig. 5b). Between the cell–cell contacts, labels are detected (Fig. 5c), suggesting the good penetration of this FLIPPER-body. Some cells have regions with less label efficiency in fluorescence as well as in electron density (Fig. 5c). This suggests an irregular distribution of HER2 in the plasma membrane, which is in agreement with the previous reports (Chung et al. 2016).

## Discussion

Genetically-encoded FLIPPER-bodies were created as an alternative for multiplex immuno-labeling with nanoparticles for CLEM. Proof-of-concepts that the single-step nanobody-based labeling is more efficient and penetrates better than traditional immuno-labeling using IgGs are given in examples of nuclear labeling as well as for endogenous plasma membrane receptor labeling, including in cell–cell contact sites. Importantly, this experimental result is a better reflection of receptor localization that would be missed by traditional CLEM or EM antibody-based labeling strategies, which does not label nuclear proteins or receptors at cell–cell contacts (see results above and Schnell et al. 2012). Improved labeling with FLIPPER-bodies may be caused by (1) better penetration into dense structures as a result of their small size (~ 75 kDa) as compared to the size of IgG antibodies (~ 150 kDa) that are linked to nanoparticles (Fig. 1a); (2) the FLIPPER-body requires a single-labeling step, the signal is not enhanced by amplification in a second step, but all target-reaching FLIPPER-bodies will have label; and (3) FLIPPER-bodies are small proteins, so it is a flexible molecule which might more easily reach the epitope in contrast to QDs or gold particles (Giepmans et al. 2005). Even compared to the bulky IgGs conjugated to a couple of small Alexa dyes, FLIPPER-bodies can reach the epitope better.

The FLIPPER-body technology represents a versatile system that can be designed to bind to any possible target, and can be provided with any possible color or peroxidase by simple molecular cloning. FLIPPER-bodies are preferentially as small as possible and easily produced and secreted in a production cell-line-like CHO cells. As nanobodies are single-chain-binding units, small, and to be generated against any possible target, the nanobody is currently the preferred binding unit. Moreover, given the versatility of nanobody applications (Beghein and Gettemans 2017) and recent establishment of a centralized nanobody database collection (ICAN; Zuo et al. 2017), the expansion of the FLIPPER-bodies has great promise. However, alternative small, single-domain-binding scaffolds capable of binding with high affinity to their target such as affibodies may also be employed in the FLIPPER-body technology (Helma et al. 2015). Note that because of the genetic encoding, molecular biology tools easily will help to refine the FLIPPER-bodies, like the continuous improvements, we have seen for fluorescent proteins and genetically-encoded biosensors (Giepmans et al. 2006; Rodriguez et al. 2017). In addition, note that, like all plasmids or hybridomas, the FLIPPER-bodies can be shared free of charge within the scientific community, as opposed to antibodies. Our future studies will assess whether the FLIPPER-bodies can also be used in Tokuyasu-like immuno-EM (Webster et al. 2008) or EPON-section labeling (Kuipers et al. 2015a) and might also be a generic tool for post-embedding labeling.

A limitation of peroxidase-based probes, like the FLIPPER-body is that in EM, labeling multiple targets cannot be distinguished, while with QDs, it is possible to distinguish multiple targets due to differences in shape (Giepmans et al. 2005). One way of using multiple targets would be using multiple fluorescent colors and later layering the images to see where each target is localized. Another option for double labeling makes use of DAB with specific incorporated lanthanides detectable with an electron energy detector (Adams et al. 2016). In this case, after the first labeling Cerium-DAB_2_, which is added and blocked, followed by the second label and black precipitates are formed with Lanthanum-DAB_2_. The different lanthanides then can be distinguished. Similarly, X-ray detection may possibly be used to discriminate between certain Lanthanides-containing DAB precipitates, which may be even multiplexed with gold particles or QDs (Scotuzzi et al. 2017).

In conclusion, FLIPPER-bodies have the potential to become a generic probe to identify proteins in CLEM with significant benefits over existing immuno-EM using IgGs, most importantly in better access to the epitope.

## Electronic supplementary material

Below is the link to the electronic supplementary material.

Supplementary material 1 (PDF 1111KB)

## References

[CR1] Adams SR, Mackey MR, Ramachandra R, Palida Lemieux SF, Steinbach P, Bushong EA, Butko MT, Giepmans BN, Ellisman MH, Tsien RY (2016). Multicolor electron microscopy for simultaneous visualization of multiple molecular species. Cell Chem Biol.

[CR2] Ariotti N, Hall TE, Rae J, Ferguson C, McMahon KA, Martel N, Webb RE, Webb RI, Teasdale RD, Parton RG (2015). Modular detection of GFP-labeled proteins for rapid screening by electron microscopy in cells and organisms. Dev Cell.

[CR3] Beghein E, Gettemans J (2017). Nanobody technology: a versatile toolkit for microscopic imaging, protein–protein interaction analysis, and protein function exploration. Front Immunol.

[CR4] Carter RE, Sorkin A (1998). Endocytosis of functional epidermal growth factor receptor-green fluorescent protein chimera. J Biol Chem.

[CR5] Chung I, Reichelt M, Shao L, Akita RW, Koeppen H, Rangell L, Schaefer G, Mellman I, Sliwkowski MX (2016). High cell-surface density of HER2 deforms cell membranes. Nat Commun.

[CR6] de Boer P, Hoogenboom JP, Giepmans BN (2015). Correlated light and electron microscopy: ultrastructure lights up!. Nat Methods.

[CR7] Giepmans BN, Deerinck TJ, Smarr BL, Jones YZ, Ellisman MH (2005). Correlated light and electron microscopic imaging of multiple endogenous proteins using quantum dots. Nat Methods.

[CR8] Giepmans BN, Adams SR, Ellisman MH, Tsien RY (2006). The fluorescent toolbox for assessing protein location and function. Science.

[CR9] Haigler H, Ash JF, Singer SJ, Cohen S (1978). Visualization by fluorescence of the binding and internalization of epidermal growth factor in human carcinoma cells A-431. Proc Natl Acad Sci USA.

[CR10] Hamers-Casterman C, Atarhouch T, Muyldermans S, Robinson G, Hamers C, Songa EB, Bendahman N, Hamers R (1993). Naturally occurring antibodies devoid of light chains. Nature.

[CR11] Hauser CT, Tsien RY (2007). A hexahistidine-Zn^2+^-dye label reveals STIM1 surface exposure. Proc Natl Acad Sci USA.

[CR12] Helma J, Cardoso MC, Muyldermans S, Leonhardt H (2015). Nanobodies and recombinant binders in cell biology. J Cell Biol.

[CR13] Kanda T, Sullivan KF, Wahl GM (1998). Histone-GFP fusion protein enables sensitive analysis of chromosome dynamics in living mammalian cells. Curr Biol.

[CR14] Kijanka M, Warnders FJ, El Khattabi M, Lub-de Hooge M, van Dam GM, Ntziachristos V, de Vries L, Oliveira S, van Bergen En Henegouwen PM (2013). Rapid optical imaging of human breast tumour xenografts using anti-HER2 VHHs site-directly conjugated to IRDye 800CW for image-guided surgery. Eur J Nucl Med Mol Imaging.

[CR15] Kijanka M, van Donselaar EG, Muller WH, Dorresteijn B, Popov-Celeketic D, El Khattabi M, Verrips CT, van Bergen En Henegouwen PM, Post JA (2017). A novel immuno-gold labeling protocol for nanobody-based detection of HER2 in breast cancer cells using immuno-electron microscopy. J Struct Biol.

[CR16] Kuipers J, de Boer P, Giepmans BN (2015). Scanning EM of non-heavy metal stained biosamples: large-field of view, high contrast and highly efficient immunolabeling. Exp Cell Res.

[CR17] Kuipers J, van Ham TJ, Kalicharan RD, Veenstra-Algra A, Sjollema KA, Dijk F, Schnell U, Giepmans BN (2015). FLIPPER, a combinatorial probe for correlated live imaging and electron microscopy, allows identification and quantitative analysis of various cells and organelles. Cell Tissue Res.

[CR18] Lam SS, Martell JD, Kamer KJ, Deerinck TJ, Ellisman MH, Mootha VK, Ting AY (2015). Directed evolution of APEX2 for electron microscopy and proximity labeling. Nat Methods.

[CR19] Loussert Fonta C, Humbel BM (2015). Correlative microscopy. Arch Biochem Biophys.

[CR20] Martell JD, Deerinck TJ, Sancak Y, Poulos TL, Mootha VK, Sosinsky GE, Ellisman MH, Ting AY (2012). Engineered ascorbate peroxidase as a genetically encoded reporter for electron microscopy. Nat Biotechnol.

[CR21] Mikhaylova M, Cloin BM, Finan K, van den Berg R, Teeuw J, Kijanka MM, Sokolowski M, Katrukha EA, Maidorn M, Opazo F, Moutel S, Vantard M, Perez F, van Bergen En Henegouwen PM, Hoogenraad CC, Ewers H, Kapitein LC (2015). Resolving bundled microtubules using anti-tubulin nanobodies. Nat Commun.

[CR22] Muyldermans S (2013). Nanobodies: natural single-domain antibodies. Annu Rev Biochem.

[CR23] Nisman R, Dellaire G, Ren Y, Li R, Bazett-Jones DP (2004). Application of quantum dots as probes for correlative fluorescence, conventional, and energy-filtered transmission electron microscopy. J Histochem Cytochem.

[CR24] Rodriguez EA, Campbell RE, Lin JY, Lin MZ, Miyawaki A, Palmer AE, Shu X, Zhang J, Tsien RY (2017). The growing and glowing toolbox of fluorescent and photoactive proteins. Trends Biochem Sci.

[CR25] Roovers RC, Vosjan MJ, Laeremans T, el Khoulati R, de Bruin RC, Ferguson KM, Verkleij AJ, van Dongen GA, van Bergen En Henegouwen PM (2011). A biparatopic anti-EGFR nanobody efficiently inhibits solid tumour growth. Int J Cancer.

[CR26] Rothbauer U, Zolghadr K, Tillib S, Nowak D, Schermelleh L, Gahl A, Backmann N, Conrath K, Muyldermans S, Cardoso MC, Leonhardt H (2006). Targeting and tracing antigens in live cells with fluorescent nanobodies. Nat Methods.

[CR27] Schnell U, Dijk F, Sjollema KA, Giepmans BN (2012). Immunolabeling artifacts and the need for live-cell imaging. Nat Methods.

[CR28] Scotuzzi M, Kuipers J, Wensveen DI, de Boer P, Hagen KC, Hoogenboom JP, Giepmans BN (2017). Multi-color electron microscopy by element-guided identification of cells, organelles and molecules. Sci Rep.

[CR29] Shaner NC, Campbell RE, Steinbach PA, Giepmans BN, Palmer AE, Tsien RY (2004). Improved monomeric red, orange and yellow fluorescent proteins derived from *Discosoma* sp. red fluorescent protein. Nat Biotechnol.

[CR30] Sokol E, Kramer D, Diercks GF, Kuipers J, Jonkman MF, Pas HH, Giepmans BN (2015). Large-scale electron microscopy maps of patient skin and mucosa provide insight into pathogenesis of blistering diseases. J Invest Dermatol.

[CR31] Webster P, Schwarz H, Griffiths G (2008). Preparation of cells and tissues for immuno EM. Methods Cell Biol.

[CR32] Yarden Y (2001). Biology of HER2 and its importance in breast cancer. Oncology.

[CR33] Zuo J, Li J, Zhang R, Xu L, Chen H, Jia X, Su Z, Zhao L, Huang X, Xie W (2017). Institute collection and analysis of nanobodies (iCAN): a comprehensive database and analysis platform for nanobodies. BMC Genom.

