# Supplementary material for: A small protein probe for correlated microscopy of endogenous proteins
Source: Histochem Cell Biol. 2018 Jan 11;149(3):261–8. doi: 10.1007/s00418-018-1632-6 (PMC5816110; doi:10.1007/s00418-018-1632-6)
Supplement: Supplementary file 1 — Supplementary material 1 (PDF 1111KB) [file 418_2018_1632_MOESM1_ESM.pdf]

Supplementary data accompanying:

'A small protein probe for correlated microscopy of endogenous proteins' by Marit A. de Beer, Jeroen Kuipers, Paul M.P. van Bergen en Henegouwen and Ben N.G. Giepmans. Published in Histochemistry and Cell Biology (2018) DOI: 10.1007/s00418-018-1632-6

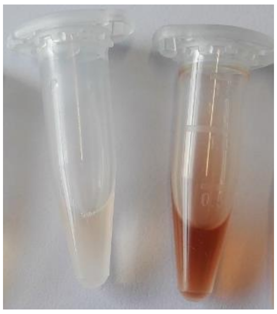

Figure S1. Secreted FLIPPER-body contains active peroxidase - Medium of CHO-K1 cells expressing SP-His_6-GPI-CFP (left; detailed in Hauser and Tsien 2007) or FLIPPER-bodies (right) was analyzed for the presence peroxidase activity. Note that following DAB conversion, His_6-GPI-CFP do not react with DAB and the FLIPPER-bodies do provide a DAB reaction.

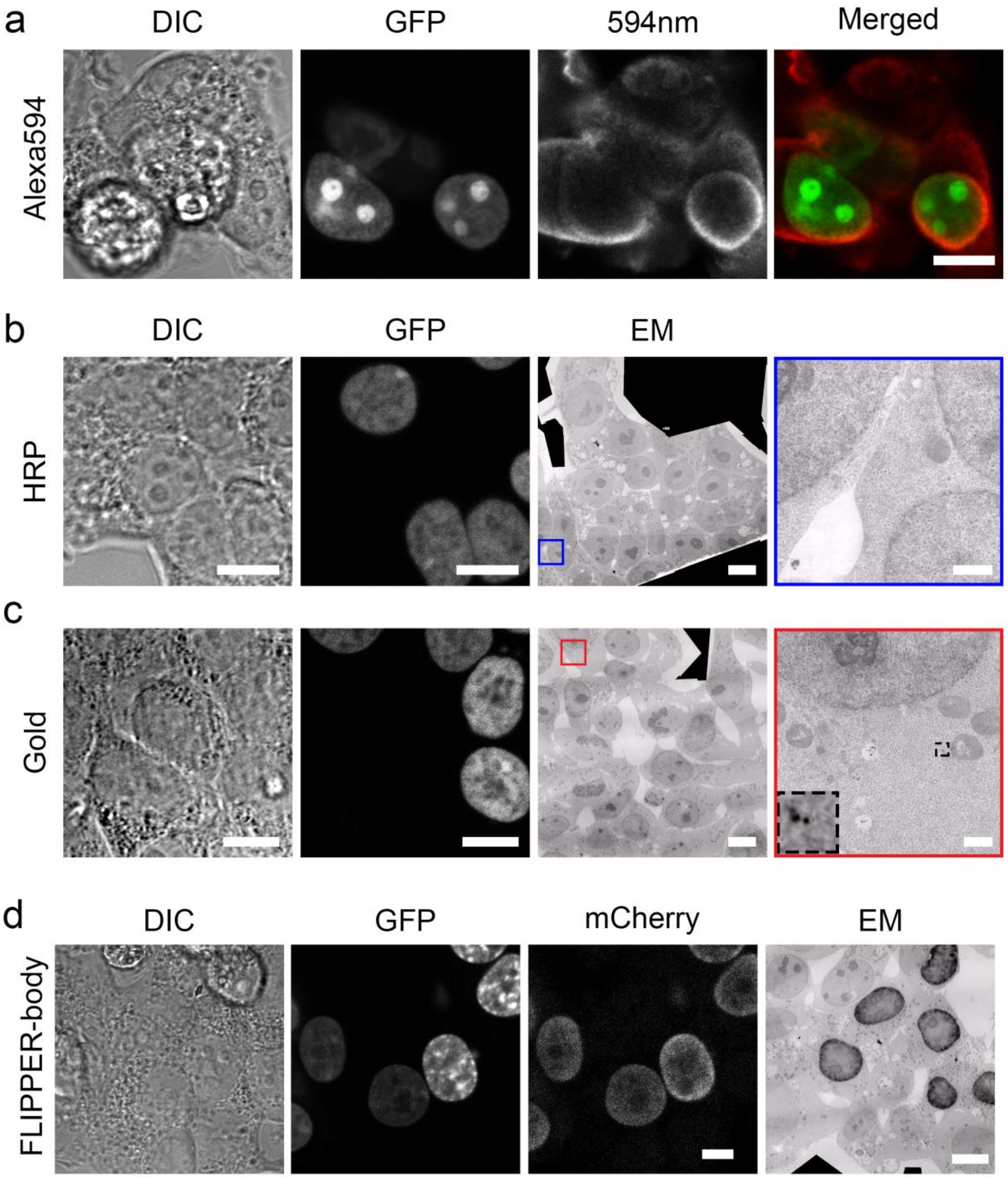

Figure S2. Detection of nuclear GFP shows limited penetration of IgG – based labeling - HEK293T cells transiently expressing H2B-GFP were fixed, permeabilized and indirectly stained with anti-GFP. (a) Secondary antibodies conjugated to Alexa Fluor594 were used to detect nuclear expressed H2B-GFP and while there is clear GFP signal in the nucleus, the A594 signal is absent from the core of the nucleus. There is also much background of the A594 in the cytoplasm. (b) Secondary antibodies conjugated to HRP were used to detect nuclear expressed H2B-GFP. Both at low as well as at high EM resolution no signal, representing HRP after DAB conversion could be detected. (c) Secondary antibodies bound to 10 nm gold label were used to detect nuclear H2B-GFP. Some background signal was observed in the cytoplasm, which can be seen in the insert. (d) FLIPPER-bodies were used

to detect nuclear H2B-GFP. Note the positive mCherry and HRP signal in LM and EM, respectively. DIC: differential interference contrast; GFP: GFP fluorescence; 594nm: secondary antibody conjugated to Alexa594; mCherry: fluorescence from FLIPPER-body; merged: GFP and indicated fluorescent signals; EM: ultrathin EM section. Bars: LM and EM overview 10 µm, EM zoom in 1 µm. High resolution EM images are available via www.nanotomy.org .

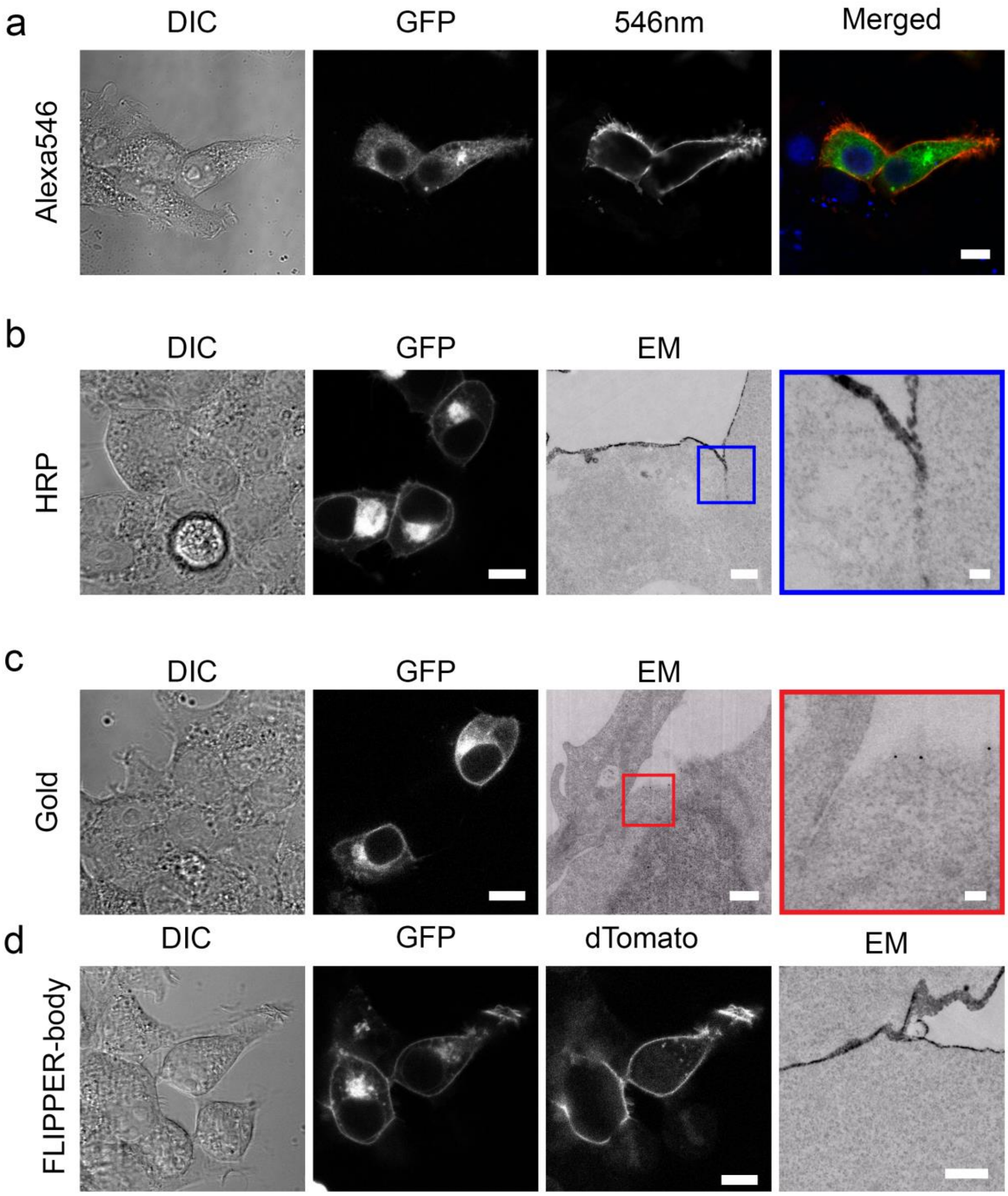

Figure S3. Detection of overexpressed EGFR using traditional antibody-based probes - HEK293T cells were transiently transfected with EGFR-GFP cDNA, fixed, pre-embedding labeled and processed for LM and EM. (a) Indirect labeling using anti-EGFR and secondary antibodies conjugated to Alexa Fluor 546 was used for detection of EGFR, showing an efficient label surrounding the cells. (b) Indirect EGFR labeling using HRP-conjugated antibodies shows an efficient labeling at the cell clusters periphery. The zoom in in EM shows the limited penetration between cell-cell contact sites. (c) EGFR detection using secondary antibodies bound to 10 nm gold seen in the EM magnification. Gold particles decorate the cells periphery, but not at cell-cell contacts sites. (d) Direct labeling of EGFR using FLIPPER-bodies showing in LM and EM high label efficiency at the cells periphery and

at cell-cell contact sites. DIC: differential interference contrast; GFP: GFP fluorescence; 546nm: secondary antibody conjugated to Alexa546; dTomato: fluorescence from FLIPPER-body; merged: GFP and indicated fluorescent signals; EM: ultrathin EM section. Bars: LM 10 µm, EM 500 nm, Zoom in 100 nm. High resolution EM images are available via www.nanotomy.org.

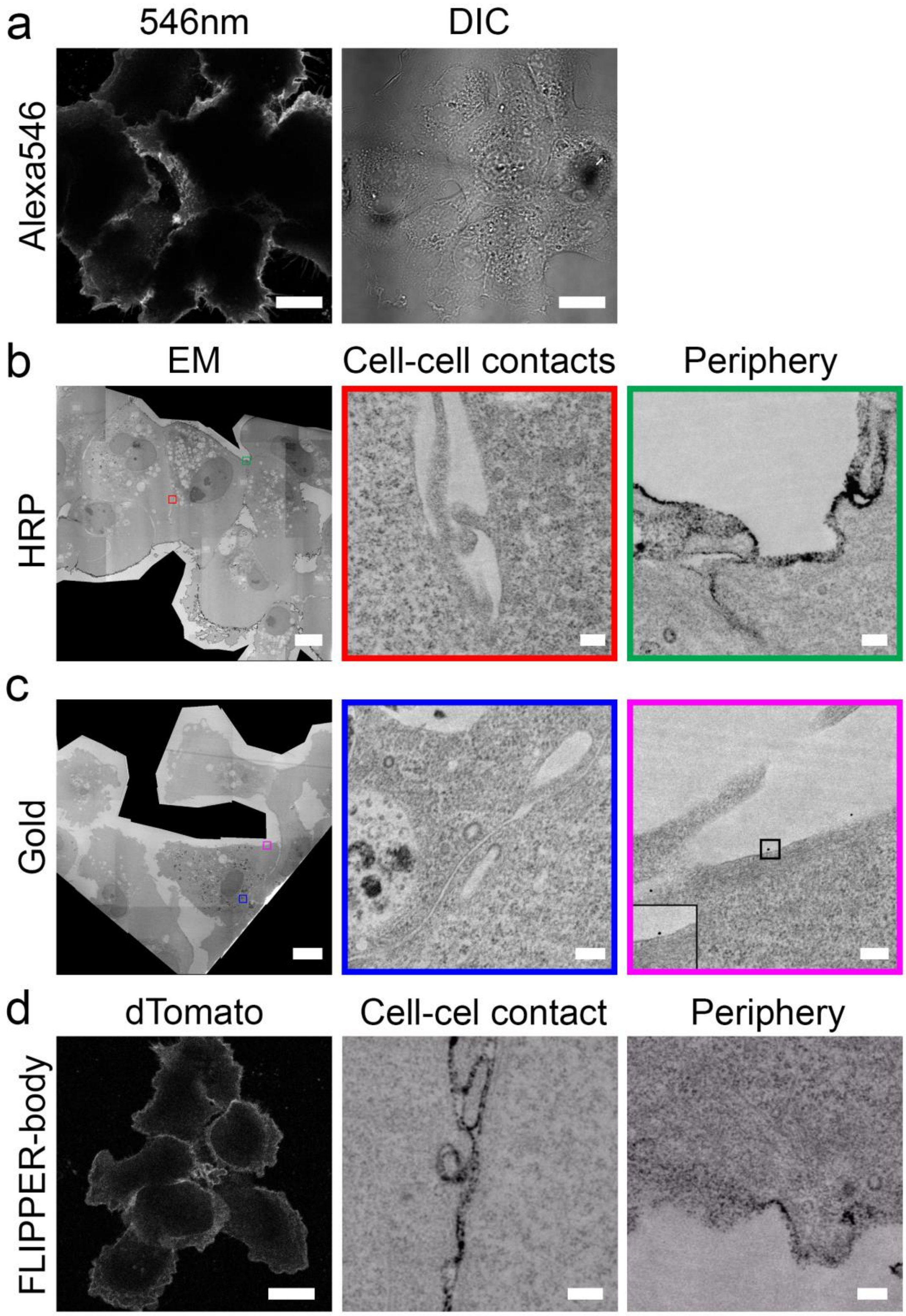

Figure S4. Detection of endogenous EGFR using different traditional labeling methods - A431 cells are fixed and labeled with anti-EGFR antibodies, detected with common used secondary antibodies conjugated to (a) Alexa546, (b), HRP, or (c), 10 nm gold particles. All cells have labeling outside the cell clusters but lack in the detection of EGFR between the

cell-cell contacts. (d) Direct labeling of EGFR with the FLIPPER-body, labeling all the cells periphery and cell-cell contact sites. DIC: differential interference contrast; 546nm: secondary antibody conjugated to Alexa546; dTomato: fluorescence from FLIPPER-body; EM: ultrathin EM section. Bars: LM 20 µm, EM overview 10 µm, zoom in 200 nm.

Supplementary data: Sequence of FLIPPER-bodies

Abbreviations and previous publication of parts of relevant cDNAs:

Signal peptide	(SP)	(Schnell et al. 2013)
6-Histidine tag	(HIS)	
Thrombin cleavage site		(Waugh 2011)
mCherry	(mCh)	(Shaner et al. 2004)
dTomato	(dTom)	(Shaner et al. 2004)
Horseradish peroxidase	(HRP)	(Kuipers et al. 2015)
Ascorbate peroxidases 2	(APEX2)	(Lam et al. 2015)
Nanobody	(NB)	
• antiGFP		(Rothbauer et al. 2006)
• antiEGFR		(Roovers et al. 2011)
• antiHER2		(Kijanka et al. 2013)

References

Kijanka M, Warnders FJ, El Khattabi M, Lub-de Hooge M, van Dam GM, Ntziachristos V, de Vries L, Oliveira S, van Bergen En Henegouwen PM (2013) Rapid optical imaging of human breast tumour xenografts using anti-HER2 VHHs site-directly conjugated to IRDye 800CW for image-guided surgery. Eur J Nucl Med Mol Imaging 40:1718-1729.

Kuipers J, van Ham TJ, Kalicharan RD, Veenstra-Algra A, Sjollema KA, Dijk F, Schnell U, Giepmans BN (2015) FLIPPER, a combinatorial probe for correlated live imaging and electron microscopy, allows identification and quantitative analysis of various cells and organelles. Cell Tissue Res 360:61-70.

Lam SS, Martell JD, Kamer KJ, Deerinck TJ, Ellisman MH, Mootha VK, Ting AY (2015) Directed evolution of APEX2 for electron microscopy and proximity labeling. Nat Methods 12:51-54.

Roovers RC, Vosjan MJ, Laeremans T, el Khoulati R, de Bruin RC, Ferguson KM, Verkleij AJ, van Dongen GA, van Bergen en Henegouwen PM (2011) A biparatopic anti-EGFR nanobody efficiently inhibits solid tumour growth. Int J Cancer 129:2013-24.

Rothbauer U, Zolghadr K, Tillib S, Nowak D, Schermelleh L, Gahl A, Backmann N, Conrath K, Muyldermans S, Cardoso MC, Leonhardt H (2006) Targeting and tracing antigens in live cells with fluorescent nanobodies. Nat Methods 3:887-9.

Schnell U, Cirulli V, Giepmans BN (2013) EpCAM: Structure and function in health and disease. Biochim Biophys Acta 1828:1989-2001.

Shaner NC, Campbell RE, Steinbach PA, Giepmans BN, Palmer AE, Tsien RY (2004) Improved monomeric red, orange and yellow fluorescent proteins derived from Discosoma sp. red fluorescent protein. Nat Biotechnol 22:1567-72.

Waugh DS (2011) An overview of enzymatic reagents for the removal of affinity tags. Protein Expr Purif 80:283-293.

Supplementary data - 'A small protein probe for correlated microscopy of endogenous proteins' *by de Beer et al. Histochem. Cell Biol. (2018) DOI: 10.1007/s00418-018-1632-6*

Sequence anti-GFP FLIPPER-body

SP-HIS-Thrombin-mCh-HRP-NB

*aagctt*cttaag*gatatc*acc**atg**gcgcccccgcaggtcctcgcgttcgggcttctgctt
M A P P Q V L A F G L L L

gccgcggcgacggcgacttttgccgcagctcaggaagaa*gatatc*atgcatcatcatcac
A A A T A T F A A A Q E E D I M H H H H

caccatcatcatctcgtaccaagaggttct*gttaacgaattc*atggtgagcaagggcgag
H H H H L V P R G S V N E F M V S K G E

gaggataacatggccatcatcaaggagttcatgcgcttcaaggtgcacatggagggctcc
E D N M A I I K E F M R F K V H M E G S

gtgaacggccacgagttcgagatcgagggcgagggcgagggccgcccctacgagggcacc
V N G H E F E I E G E G E G R P Y E G T
cagaccgccaagctgaaggtgaccaagggtggccccctgcccttcgcctgggacatcctg
Q T A K L K V T K G G P L P F A W D I L

tcccctcagttcatgtacggctccaaggcctacgtgaagcaccccgccgacatccccgac
S P Q F M Y G S K A Y V K H P A D I P D

tacttgaagctgtccttccccgagggcttcaagtgggagcgcgtgacgaacttcgaggac
Y L K L S F P E G F K W E R V T N F E D

ggcggcgtggtgaccgtgacccaggactcctccctgcaggacggcgagttcatctacaag
G G V V T V T Q D S S L Q D G E F I Y K

gtgaagctgcgcggcaccaacttcccctccgacggccccgtaatgcagaagaagaccatg
V K L R G T N F P S D G P V M Q K K T M

ggctgggaggcctcctccgagcggatgtaccccgaggacggcgccctgaagggcgagatc
G W E A S S E R M Y P E D G A L K G E I

aagcagaggctgaagctgaaggacggcggccactacgacgctgaggtcaagaccacctac
K Q R L K L K D G G H Y D A E V K T T Y

aaggccaagaagcccgtgcagctgcccggcgcctacaacgtcaacatcaagttggacatc
K A K K P V Q L P G A Y N V N I K L D I

acctcccacaacgaggactacaccatcgtggaacagtacgaacgcgccgagggccgccac
T S H N E D Y T I V E Q Y E R A E G R H

tccaccggcggcatggacgagctgtacaag*ggatcc*atgcagttaacccctacattctac
S T G G M D E L Y K G S M Q L T P T F Y

gacaatagctgtcccaacgtgtccaacatcgttcgcgacacaatcgtcaacgagctcaga
D N S C P N V S N I V R D T I V N E L R

tccgatcccaggatcgctgcttcaatattacgtctgcacttccatgactgcttcgtgaat
S D P R I A A S I L R L H F H D C F V N

ggttgcgacgctagcatattactggacaacaccaccagtttccgcactgaaaaggatgca
G C D A S I L L D N T T S F R T E K D A

ttcgggaacgctaacagcgccaggggctttccagtgatcgatcgcatgaaggctgccgtt

F G N A N S A R G F P V I D R M K A A V

gagtcagcatgcccacgaacagtcagttgtgcagacctgctgactatagctgcgcaacag

E S A C P R T V S C A D L L T I A A Q Q

agcgtgactcttgcaggcggaccgtcctggagagtgccgctcggtcgacgtgactcccta

S V T L A G G P S W R V P L G R R D S L

caggcattcctagatctggccaacgccaacttgcctgctccattttcaccctgccccag

Q A F L D L A N A N L P A P F F T L P Q

ctgaaggatagctttagaaacgtgggtctgaatcgctcaagtgaccttgtggctctgtcc

L K D S F R N V G L N R S S D L V A L S

ggaggacacacgtttggaaagaaccagtgtaggttcatcatggataggctctacaatttc

G G H T F G K N Q C R F I M D R L Y N F

agcaacactgggttacctgaccccacgctgaacactacgtatctccagacactgagaggc

S N T G L P D P T L N T T Y L Q T L R G

ttgtgcccactgaatggcaacctcagtgcactagtggactttgatctgcggaccccaacc

L C P L N G N L S A L V D F D L R T P T

atcttcgataacaagtactatgtgaatctagaggagcagaaaggcctgatacagagtgat

I F D N K Y Y V N L E E Q K G L I Q S D

caagaactgtttagcagtccaaacgccactgacaccatcccactggtgagaagttttgct

Q E L F S S P N A T D T I P L V R S F A

aactctactcaaaccttctttaacgccttcgtggaagccatggaccgtatgggtaacatt

N S T Q T F F N A F V E A M D R M G N I

acccctctgacgggtacccaaggccagattcgtctgaactgcaaagtggtcaacagcaac

T P L T G T Q G Q I R L N C K V V N S N

tctaggggaggagga*gcggccgc*tatggatcaagtccaactggtggagtctggtggcgct

S R G G G A A A M D Q V Q L V E S G G A

ttggtgcagccaggtggctctctgcgtttgtcctgtgccgcttctggcttcccagtgaac

L V Q P G G S L R L S C A A S G F P V N

cgctattccatgcgctggtatcgccaggctccaggcaaagagcgtgagtgggtagccggt

R Y S M R W Y R Q A P G K E R E W V A G

atgtccagcgcgggtgatcgtagctcctatgaagactccgtgaagggccgtttcaccatc

M S S A G D R S S Y E D S V K G R F T I

agccgtgacgatgcccgtaacacggtgtatctgcaaatgaacagcttgaaacctgaagat

S R D D A R N T V Y L Q M N S L K P E D

acggccgtgtattactgtaatgtgaacgtgggcttcgagtattggggccaaggcacccag

T A V Y Y C N V N V G F E Y W G Q G T Q

gtcaccgtctccagc**taa***accggt*

V T V S S *

Sequence anti-EGFR FLIPPER-body

SP-NB-dTom-APEX2-HIS

```
gatatcaagctt**atg**gcgcccccgcaggtcctcgcgttcgggcttctgcttgccgcggcg
             M  A  P  P  Q  V  L  A  F  G  L  L  L  A  A  A

Acggcgacttttgccgcagctcaggaagaaaccggt (Nanobody) gaattcatggtg

 T  A  T  F  A  A  A  Q  E  E  T  G  Nanobody     E  F  M  V

agcaagggcgaggaggtcatcaaagagttcatgcgcttcaaggtgcgcatggagggctcc
 S  K  G  E  E  V  I  K  E  F  M  R  F  K  V  R  M  E  G  S

atgaacggccacgagttcgagatcgagggcgagggcgagggccgcccctacgagggcacc
 M  N  G  H  E  F  E  I  E  G  E  G  E  G  R  P  Y  E  G  T

cagaccgccaagctgaaggtgaccaagggcggccccctgcccttcgcctgggacatcctg
 Q  T  A  K  L  K  V  T  K  G  G  P  L  P  F  A  W  D  I  L

tcccccagttcatgtacggctccaaggcgtacgtgaagcaccccgccgacatccccgat
 S  P  Q  F  M  Y  G  S  K  A  Y  V  K  H  P  A  D  I  P  D

tacaagaagctgtccttccccgagggcttcaagtgggagcgcgtgatgaacttcgaggac
 Y  K  K  L  S  F  P  E  G  F  K  W  E  R  V  M  N  F  E  D

ggcggtctggtgaccgtgacccaggactcctccctgcaggacggcacgctgatctacaag
 G  G  L  V  T  V  T  Q  D  S  S  L  Q  D  G  T  L  I  Y  K

gtgaagatgcgcggcaccaacttcccccccgacggccccgtaatgcagaagaagaccatg
 V  K  M  R  G  T  N  F  P  P  D  G  P  V  M  Q  K  K  T  M

ggctgggaggcctccaccgagcgcctgtaccccgcgacggcgtgctgaagggcgagatc
 G  W  E  A  S  T  E  R  L  Y  P  R  D  G  V  L  K  G  E  I

caccaggccctgaagctgaaggacggcggccactacctggtggagttcaagaccatctac
 H  Q  A  L  K  L  K  D  G  G  H  Y  L  V  E  F  K  T  I  Y

atggccaagaagcccgtgcaactgcccggctactactacgtggacaccaagctggacatc
 M  A  K  K  P  V  Q  L  P  G  Y  Y  Y  V  D  T  K  L  D  I

acctcccacaacgaggactacaccatcgtggaacagtacgagcgctccgagggccgccac
 T  S  H  N  E  D  Y  T  I  V  E  Q  Y  E  R  S  E  G  R  H

cacctgttcctgtacggcatggacgagctgtacaagggatccggaaagtcttacccaact
 H  L  F  L  Y  G  M  D  E  L  Y  K  G  S  G  K  S  Y  P  T

gtgagtgctgattaccaggacgccgttgagaaggcgaagaagaagctcagaggcttcatc
 V  S  A  D  Y  Q  D  A  V  E  K  A  K  K  K  L  R  G  F  I

gctgagaagagatgcgctcctctaatgctccgtttggcattccactctgctggaaccttt
 A  E  K  R  C  A  P  L  M  L  R  L  A  F  H  S  A  G  T  F

gacaagggcacgaagaccggtggacccttcggaaccatcaagcaccctgccgaactggct
 D  K  G  T  K  T  G  G  P  F  G  T  I  K  H  P  A  E  L  A

cacagcgctaacaacggtcttgacatcgctgttaggcttttggagccactcaaggcggag
```

```
 H   S   A   N   N   G   L   D   I   A   V   R   L   L   E   P   L   K   A   E

ttccctattttgagctacgccgatttctaccagttggctggcgttgttgccgttgaggtc
 F   P   I   L   S   Y   A   D   F   Y   Q   L   A   G   V   V   A   V   E   V

acgggtggacctaaggttccattccaccctggaagagaggacaagcctgagccaccacca
 T   G   G   P   K   V   P   F   H   P   G   R   E   D   K   P   E   P   P   P

gagggtcgcttgcccgatcccactaagggttctgaccatttgagagatgtgtttggcaaa
 E   G   R   L   P   D   P   T   K   G   S   D   H   L   R   D   V   F   G   K

gctatggggcttactgaccaagatatcgttgctctatctggggggtcacactattggagct
 A   M   G   L   T   D   Q   D   I   V   A   L   S   G   G   H   T   I   G   A

gcacacaaggagcgttctggatttgagggtccctggacctctaatcctcttattttcgac
 A   H   K   E   R   S   G   F   E   G   P   W   T   S   N   P   L   I   F   D

aactcatacttcacggagttgttgagtggtgagaaggaaggtctccttcagctaccttct
 N   S   Y   F   T   E   L   L   S   G   E   K   E   G   L   L   Q   L   P   S

gacaaggctcttttgtctgaccctgtattccgccctctcgttgacaaatatgcagcggac
 D   K   A   L   L   S   D   P   V   F   R   P   L   V   D   K   Y   A   A   D

gaagatgccttctttgctgattacgctgaggctcaccaaaagctttccgagcttgggttt
 E   D   A   F   F   A   D   Y   A   E   A   H   Q   K   L   S   E   L   G   F

gctgatgccgcggccgctcatcatcatcaccaccattaactcgagtctaga
 A   D   A   A   A   A   H   H   H   H   H   H   *
```

Sequence anti-HER2 FLIPPER-body

SP-NB-dTom-APEX2-HIS

*gatatcaagctt***atg**gcgcccccgcaggtcctcgcgttcgggcttctgcttgccgcggcg
M A P P Q V L A F G L L L A A A

Acggcgacttttgccgcagctcaggaagaa*accggt (NB) gaattc*atggtgagcaagggcgag

T A T F A A A Q E E T G NB E F M V S K G E

gaggtcatcaaagagttcatgcgcttcaaggtgcgcatggagggctccatgaacggccac
E V I K E F M R F K V R M E G S M N G H

gagttcgagatcgagggcgagggcgagggccgcccctacgagggcacccagaccgccaag
E F E I E G E G E G R P Y E G T Q T A K

ctgaaggtgaccaagggcggccccctgcccttcgcctgggacatcctgtccccccagttc
L K V T K G G P L P F A W D I L S P Q F

atgtacggctccaaggcgtacgtgaagcaccccgccgacatccccgattacaagaagctg
M Y G S K A Y V K H P A D I P D Y K K L

tccttccccgagggcttcaagtgggagcgcgtgatgaacttcgaggacggcggtctggtg
S F P E G F K W E R V M N F E D G G L V

accgtgacccaggactcctccctgcaggacggcacgctgatctacaaggtgaagatgcgc
T V T Q D S S L Q D G T L I Y K V K M R

ggcaccaacttcccccccgacggccccgtaatgcagaagaagaccatgggctgggaggcc
G T N F P P D G P V M Q K K T M G W E A

tccaccgagcgcctgtaccccgcgacggcgtgctgaagggcgagatccaccaggccctg
S T E R L Y P R D G V L K G E I H Q A L

aagctgaaggacggcggccactacctggtggagttcaagaccatctacatggccaagaag
K L K D G G H Y L V E F K T I Y M A K K

cccgtgcaactgcccggctactactacgtggacaccaagctggacatcacctcccacaac
P V Q L P G Y Y Y V D T K L D I T S H N

gaggactacaccatcgtggaacagtacgagcgctccgagggccgccaccacctgttcctg
E D Y T I V E Q Y E R S E G R H H L F L

tacggcatggacgagctgtacaag*ggatcc*ggaaagtcttacccaactgtgagtgctgat
Y G M D E L Y K G S G K S Y P T V S A D

taccaggacgccgttgagaaggcgaagaagaagctcagaggcttcatcgctgagaagaga
Y Q D A V E K A K K K L R G F I A E K R

tgcgctcctctaatgctccgtttggcattccactctgctggaacctttgacaagggcacg
C A P L M L R L A F H S A G T F D K G T

aagaccggtggacccttcggaaccatcaagcaccctgccgaactggctcacagcgctaac
K T G G P F G T I K H P A E L A H S A N

aacggtcttgacatcgctgttaggcttttggagccactcaaggcggagttccctattttg
N G L D I A V R L L E P L K A E F P I L

agctacgccgatttctaccagttggctggcgttgttgccgttgaggtcacgggtggacct
S Y A D F Y Q L A G V V A V E V T G G P

aaggttccattccaccctggaagagaggacaagcctgagccaccaccagagggtcgcttg
K V P F H P G R E D K P E P P P E G R L

cccgatcccactaagggttctgaccatttgagagatgtgtttggcaaagctatggggctt
P D P T K G S D H L R D V F G K A M G L

actgaccaagatatcgttgctctatctggggggtcacactattggagctgcacacaaggag
T D Q D I V A L S G G H T I G A A H K E

cgttctggatttgagggtccctggacctctaatcctcttatttcgacaactcatacttc
R S G F E G P W T S N P L I F D N S Y F

acggagttgttgagtggtgagaaggaaggtctccttcagctaccttctgacaaggctctt
T E L L S G E K E G L L Q L P S D K A L

ttgtctgaccctgtattccgccctctcgttgacaaatatgcagcggacgaagatgccttc
L S D P V F R P L V D K Y A A D E D A F

tttgctgattacgctgaggctcaccaaaagctttccgagcttgggtttgctgatgcc*gcg*
F A D Y A E A H Q K L S E L G F A D A A

*gccgc*tcatcatcatcaccaccat**taa***ctcgagtctaga*
A A H H H H H H *
